# Low self-esteem is related to depression and anxiety during recovery from an ectopic pregnancy

**DOI:** 10.1186/s12905-021-01467-2

**Published:** 2021-09-08

**Authors:** Sonia Hasani, Eindra Aung, Mojgan Mirghafourvand

**Affiliations:** 1 Student Research Committee, Faculty of Nursing and Midwifery, Tabriz University of Medical Sciences, Tabriz, Iran; 2 Department of Clinical Pharmacology and Toxicology, St Vincent’s Clinical School, University of New South Wales, Kensington, Australia; 3 Midwifery Department, Social Determinants of Health Research Center, Faculty of Nursing and Midwifery, Tabriz University of Medical Sciences, Tabriz, Iran

**Keywords:** Mental health, Self-esteem, Ectopic pregnancy, Women, Depression, Anxiety

## Abstract

**Background:**

Considering the increasing incidence of ectopic pregnancy and the negative effects of pregnancy loss on mental health, this study aimed to determine the status of mental health in women with ectopic pregnancy and examine its relationship with their self-esteem.

**Methods:**

This was a cross-sectional study on 150 women (with a 100% response rate) hospitalized for ectopic pregnancy et al.-Zahra hospital in Tabriz, Iran, during 2018–2019, and recruited via convenience sampling. Data were collected using the General Health Questionnaire-28, which has four subscales (overall score range: 0 to 84; subscale score range: 0 to 21 with a lower score indicating a better mental state), and Rosenberg Self-Esteem Scale (score range: − 10 to + 10 with a higher score indicating higher self-esteem). To determine the association between self-esteem and mental health, independent t-tests, and multivariable logistic regression were used.

**Results:**

The response rate was 100%. The mean score (SD) of participants’ mental health was 31.4 (8.5), and that of self-esteem was 4.5 (3.80). The percentage of participants who were considered as having mental distress (i.e., overall GHQ-28 score ≥ 24) was 76%. Among the subscales of mental health, social dysfunction was the most prevalent (observed in 100% of the participants), followed by somatic symptoms (79.3%). Lower self-esteem was significantly associated with overall mental distress (odds ratio (OR): 0.74; 95% confidence interval (95% CI): 0.64–0.87; *P* < 0.001), depression (OR: 0.70; 95% CI: 0.60–0.80; *P* < 0.001) and anxiety/insomnia (OR: 0.76; 95% CI: 0.66–0.87; *P* < 0.001).

**Discussion:**

Mental distress was common among women with ectopic pregnancy. This study is the first to examine the relationship between self-esteem and mental health among women with ectopic pregnancy and highlights the important role of self-esteem in mental wellbeing among those women.

## Background

Pregnancy and the postpartum period are considered the most important events in a woman’s life, and can also be among the most stressful life events regardless of the type of pregnancy or its outcome [1]. Pregnancy loss, resulting from events such as medical termination, miscarriage, stillbirth, and ectopic pregnancy (EP), is commonly associated with grief and stress following the event [2]. Across the world, about 10% of pregnant women and 13% of women who have just given birth experience a mental disorder, most commonly depression. This is higher in developing countries, with 15.6% during pregnancy and 19.8% postpartum [3].

Ectopic pregnancy accounts for 1–2% of all pregnancies in various countries including the United States, and its incidence is increasing worldwide [4, 5]. The prevalence of ectopic pregnancy is 0.5–1.5% of all first-trimester pregnancies in the United States. Approximately 95% of ectopic pregnancies occur in different parts of the fallopian tube, and the remaining 5% are non-tubular and located in the ovaries, peritoneal cavity, cervix, or a previous cesarean section scar [6]. Ectopic pregnancies, although they account for a small proportion of pregnancies, indirectly account for 6% of all pregnancy-related deaths [7]. Numerous studies have determined possible risk factors for ectopic pregnancy, including age, history of ectopic pregnancy, previous and current use of intra-uterine devices (IUDs), smoking, history of pelvic surgery, history of appendectomy, oral contraceptives (OCPs), Levonorgestrel—Emergency Contraceptive (LNG-EC), history of pelvic inflammatory disease, female sterilization, and infertility history [8–16]. Various studies have shown that pregnancy loss including ectopic pregnancy can negatively affect the mental health of mothers [19–21].

Mental health is a state of well-being in which an individual is aware of their potential abilities, can cope with natural life stresses, works productively and fruitfully, and can contribute to their community [17]. Women who have experienced stillbirth or child death are found to have lower self-esteem [22]. Self-esteem can be explained through three interrelated concepts: (1) positive or negative feelings about oneself in general, (2) feelings of self-worth in particular, and (3) self-evaluation of one’s own characteristics, abilities, and attributes [18]. A study among post-partum mothers showed that those with low self-esteem were 39 times more likely to present with high-level depressive symptoms than those with high self-esteem, and that high self-esteem may compensate for the effects of stressful events [23]. Thus, self-esteem may be a critical internal resource, sustaining women during turbulent times [24–26]. Indeed, women with higher self-esteem have the internal strength to redirect or reinterpret stress and resist interpreting it negatively [26–28].

In recent years, an increase has been observed in the incidence of ectopic pregnancies [29]. Ectopic pregancy has a profound impact not only on physical health, often requiring urgent surgical treatment and carrying a risk of future infertility, but also on mental health, with previous research indicating a higher risk of post-traumatic stress disorder and suicide [30, 31]. However, there are limited studies assessing the psychological impact of ectopic pregnancy and no study to date has examined the relationship between self-esteem and mental health in women with ectopic pregnancy. It seems that the impact of ectopic pregnancy on women’s mental health can differ according to their self-esteem. The purpose of the current study was to determine the status of mental health (mental distress and symptoms of anxiety, insomnia, social dysfunction and depression) among women with ectopic pregnancy and the relationship of self-esteem and mental health in these women after losing their pregnancy. The results of the current study are expected to contribute to the body of knowledge of the psychological impact of experiencing an ectopic pregnancy, and also to the development of interventions targeting the promotion of self-esteem and mental health in women who suffer an ectopic pregnancy.

## Methods

### Study design and participants

Participants in this cross-sectional study were 150 women with EP, referred to the surgical ward of Al-Zahra hospital of Tabriz, Iran in 2018 and 2019. The inclusion criteria were women with ectopic pregnancy, according to their medical records, who were willing to participate in the study (100% response rate). Women were excluded from the study if they had severe psychological problems, personality disorders, and psychiatric disorders; had experienced mental and psychological problems such as losing their loved ones in the last 6 months; had a history of taking psychiatric medication, including anti-anxiety and anti-depressant drugs before pregnancy; had a history of psychedelic drug use; and had a previous history of ectopic pregnancy with the loss of both of their fallopian tubes. Ten women with a previous ectopic pregnancy were excluded.

The sample size in this study was calculated using G-Power software based on the largest standard deviation of self-esteem (0.66) from the study by Wonch Hill, with regard to 0.05 precision around the mean (3.32) [22], and the standard deviation of the mental health (11.5) from the study by Hasanpour, et al. with regard to 0.1 precision around the mean (29.7) [32]. The sample size required for a test power of 90% was 123 and 117 respectively. For the present study, considering the probable dropouts, 150 women were recruited into the study.

Convenience sampling was used. All participants were informed about the purpose of the study and assessed for eligibility according to the inclusion and exclusion criteria. If they were eligible to participate in the study and were willing to participate, written informed consent was obtained from them.

### Data collection

Using a structured questionnaire, data were collected from the study participants in a face-to-face interview after completing their treatment for ectopic pregnancy et al.-Zahra Hospital, Tabriz. Data on socio-demographic and obstetric characteristics were collected, including questions on age, age of marriage, spouse’s age, couple’s level of education and job and adequacy of income, risk factors of EP, contraceptive method used, type of hospitalization, and the method of diagnosis and treatment provided.

Persian versions of the Rosenberg Self-Esteem Scale (RSES) and Goldberg’s General Health Questionnaire (GHQ) were used in this study. The RSES is a 10-item instrument that measures self-esteem. The reliability and validity of the Persian version of RSES were confirmed in three studies in Iran with Cronbach’s alpha coefficient ranging from 0.69 to 0.84 [33–35]. The version used in this study includes five positively stated items and five negatively stated items, rated on a two-point scale with the item score of +1 for an “Agree” response and −1 for a “Disagree” response, the total score ranging from “− 10” to “+10”, and the higher score indicating higher self-esteem.

The 28-item GHQ (GHQ-28) was used to assess participants’ current mental health. Ebrahimi et al. examined the reliability and validity of the Persian version of the GHQ-28 in Iran and recommended that the questionnaire can be used as a screening tool in epidemiological studies of mental disorders [36]. It has also been tested among women with infertility in Iran [37]. The GHQ-28 has four subscales, which assess somatic symptoms (Items 1–7), anxiety/insomnia (Items 8–14), social dysfunction (Items 15–21), and severe depression (Items 22–28). Each subscale has 7 items, and each item is scored on a four-point Likert scale, with the item score ranging from 0 to 3, the subscale score ranging from 0 to 21, the overall score ranging from 0 to 84, and a lower score indicating a better mental state. Participants who scored 6 or more in each subscale and 24 or more on the overall scale are considered to be suffering from mental distress.

### Statistical analyses

Data were analyzed using SPSS for Windows (version 24; SPSS Inc., Chicago, IL., USA). To describe the socio-demographic characteristics, obstetric history, mental health and self-esteem, descriptive statistics were used consisting of frequency, percentage, mean, and standard deviation. The Pearson correlation test and independent t-test were used to explore the relationship of self-esteem with mental health and its subscales including somatic symptoms, anxiety/insomnia, social dysfunction, and severe depression. To confirm this relationship, logistic regression was used, adjusting for age and socio-economic status.

## Results

The response rate of the participants was 100%. The mean age in years (SD), age of marriage, and age of the spouse of women participating in this study were 29.9 (5.83), 21.5 (4.91), and 34.7 (5.84) respectively. More than one-third (36.7%) of participants were college graduates and three-quarters of them (74.7%) housewives. Also, 35.3% of spouses were college graduates and more than half (60.0%) of the men had free jobs, such as taxi driver, barber, tailor, and other jobs that do not have any insurance. More than two-thirds (68.0%) of participants stated that their family income for living expenses was adequate to some extent. Nearly all (91.3%) of participants had only one marriage. Before their ectopic pregnancy, 52.6% and 77.3% have experienced at least one pregnancy and one delivery respectively, and one-fifth of participants had experienced an abortion. None of the patient characteristics or obstetric history differ between different mental health states (Table 1). Among the women who have participated in this study, 52% of them did not use any contraceptive methods. The mean (SD) of gestational age was 5.84 (1.49), and 42% of them were treated with methotrexate. Both of the fallopian tubes and the ovaries were retained in 70.7% of the participants, and only one fallopian tube and both of the ovaries were retained in 27.3% of them. The number of average days spent in the hospital ranged from 2 to 7 days.

**Table 1:** Socio-demographic and obstetric characteristics of participants (n = 150)

Characteristics	All participants	Mental distress		*P* value ^#^
		Yes*	No	
*Age (years)*				0.15
< 25	32 (21.3)	26 (22.8)	6 (16.7)	
25–35	93 (62.0)	66 (57.9)	27 (75.0)	
> 35	25 (16.7)	22 (19.3)	3 (8.3)	
Mean (SD)	29.9 (5.83)	30.1 (6.06)	29.4 (5.08)	0.52
*Age of marriage (years)*				
Mean (SD)	21.5 (4.91)	21.5 (5.01)	21.4 (4.62)	0.86
*Spouse’s age (years)*				
Mean (SD)	34.7 (5.84)	34.8 (6.31)	34.4 (4.03)	0.71
*Education*				0.80
Illiterate	2 (1.3)	2 (1.8)	0 (0)	
Elementary	22 (14.7)	17 (14.9)	5 (13.9)	
Intermediate	19 (12.7)	13 (11.4)	6 (16.7)	
Secondary	3 (2.0)	3 (2.6)	0 (0)	
Diploma	49 (32.7)	38 (33.3)	11 (30.6)	
College	55 (36.7)	41 (36.0)	14 (38.9)	
*Occupation*				0.73
Housewife	112 (74.7)	85 (74.5)	27 (75.0)	
Working outdoor	29 (19.3)	23 (20.2)	6 (16.7)	
Working indoor	8 (5.3)	5 (4.4)	3 (8.3)	
Retired	1 (0.7)	1 (0.9)	0 (0)	
*Spouse’s education*				0.48
Elementary	18 (12.0)	16 (14.0)	2 (5.6)	
Intermediate	29 (19.3)	19 (16.7)	10 (27.8)	
Secondary	5 (3.3)	4 (3.5)	1 (2.8)	
Diploma	45 (30.0)	35 (30.7)	10 (27.8)	
College	53 (35.3)	40 (35.1)	13 (36.1)	
*Spouse’s occupation*				0.32
Free job	90 (60.0)	68 (59.6)	22 (61.1)	
Employee	40 (26.7)	33 (28.9)	7 (19.4)	
Employer	20 (13.3)	13 (11.4)	7 (19.4)	
*Adequacy of income*				0.84
Adequate	102 (68.0)	78 (68.4)	24 (66.7)	
Inadequate	48 (32.0)	36 (31.6)	12 (33.3)	
*Number of marriages*				0.55
1	137 (91.3)	105 (92.1)	32 (88.9)	
2	13 (8.7)	9 (7.9)	4 (11.1)	
*Number of pregnancies*				0.87
0	32 (21.3)	23 (20.2)	9 (25.0)	
1	47 (31.3)	37 (32.5)	10 (27.8)	
2	41 (27.3)	32 (28.1)	9 (25.0)	
≥ 3	30 (20.0)	22 (19.3)	8 (22.2)	
*Number of deliveries*				0.96
0	45 (30.0)	35 (31.0)	10 (28.6)	
1	71 (47.3)	54 (47.8)	17 (48.6)	
≥ 2	32 (21.3)	24 (21.2)	8 (22.9)	
*Number of abortions*				0.77
0	101 (67.3)	75 (65.8)	26 (72.2)	
1	30 (20.0)	24 (21.1)	6 (16.7)	
≥ 2	19 (12.7)	15 (13.2)	4 (11.1)	
*Ectopic pregnancy history*				0.91
Yes	20 (13.3)	15 (13.2)	5 (13.9)	
No	130 (86.7)	99 (86.8)	31 (86.1)	

Data are presented in N (%) unless otherwise indicated

*Overall GHQ-28 score  ≥ 24

^#^T-test for mean differences and chi-square test for differences in proportions

The mean overall GHQ-28 score (SD) of the mental health of participants was 31.4 (8.5) with their scores ranging from 14 to 50. Among the mental health subscales, the highest mean score (SD) was observed for the social dysfunction subscale [11.3 (1.6)], and the lowest score for the depression subscale [4.3 (3.9)]. The percentage of participants who were considered as having mental distress (i.e., overall GHQ-28 score ≥ 24) was 76%. Social dysfunction was the most prevalent (being observed in 100% of the participants), and somatic symptoms were the second most prevalent (79.3%). The mean score of self-esteem (SD) of participants was 4.5 (3.8), with their scores ranging from − 10 to + 10. Based on the results of the Pearson correlation test, self-esteem had a significant negative correlation with the total score of mental health (r =  − 0.40), anxiety (r =  − 0.25), social dysfunction (r =  − 0.27) and depression (r =  − 0.65) (Table 2).

**Table 2:** Mental health status and its subdomains and self-esteem status in women with Ectopic Pregnancy (n = 150)

Variable	Mean (SD*)	Obtainable score range	Obtained score range	Correlation with self-esteem
				r (*P*)^†^
Overall mental health score	31.4 (8.5)	0–84	14–50	− 0.40 (< 0.0001)
Somatic symptoms	7.9 (2.7)	0–21	2–14	− 0.11 (0.181)
Anxiety/insomnia	7.8 (4.1)	0–21	0–17	− 0.25 (0.002)
Social dysfunction	11.3 (1.6)	0–21	6–15	− 0.27 (0.001)
Depression	4.3 (3.9)	0–21	0–16	− 0.65 (< 0.0001)
Self-esteem	4.5 (3.8)	− 10 to + 10	− 10 to + 10	–

* Standard Deviation; ^†^The results are based on Pearson Correlation test

According to findings from the independent t-test, mean RSES (self-esteem) scores of participants with mental distress were found to be significantly different from the mean RSES scores of those without mental distress (t(df) = 3.57(148), *P* < 0.001). The mean RSES scores of participants with anxiety/insomnia were significantly lower than those without anxiety/insomnia (t(df) = 3.79(148), *P* < 0.001), and the mean RSES scores of participants with depression were significantly lower than those without depression (t(df) = 6.31(148), *P* < 0.001). There was no significant difference between women with and without somatic symptoms in terms of RSES scores (t(df) = 1.22(148), *P* = 0.222) (Table 3).

**Table 3:** The prevalence of mental distress and its relationship with self-esteem (n = 150)

Characteristics	Number (%)	Mean (SD*) of self-esteem score	*P* value^†^
*Mental distress*			< 0.001
Yes	114 (76.0)	3.9 (3.8)	
No	36 (24.0)	6.4 (3.3)	
*Somatic symptoms*			0.222
Yes	119 (79.3)	4.4 (3.9)	
No	31 (20.7)	5.3 (3.4)	
*Anxiety/insomnia*			< 0.001
Yes	101 (67.3)	3.8 (4.0)	
No	49 (32.7)	6.2 (2.9)	
*Social dysfunction*			
Yes	150 (100.0)	4.5 (3.8)	
No	0 (0.0)	0.0 (0.0)	
*Depression*			< 0.001
Yes	53 (35.3)	2.2 (3.9)	
No	97 (64.7)	5.8 (3.0)	

* Standard deviation; ^†^The results are based on independent t-test

Univariate logistic regression analysis showed that for a one unit increase in RSES (self-esteem), there was a decrease in the odds of having mental distress (i.e., overall GHQ-28 score ≥ 24) by 20%: unadjusted odds ratio (OR) of 0.8 with 95% confidence interval (CI) of 0.70 to 0.91 (*P* = 0.001) (Table 4). This association was found to be more statistically significant in the multivariable logistic regression model with an adjusted OR of 0.74 (95% CI: 0.64–0.87; *P* < 0.001). Self-esteem also had a statistically significant effect (*P* < 0.001) on having anxiety/insomnia (OR: 0.76; 95% CI: 0.66–0.87), and depression (OR: 0.70; 95% CI: 0.60–0.80), but had no such effect on somatic symptoms (OR: 0.88; 95% CI: 0.77–1.01; *P* = 0.062).

**Table 4:** Odds ratios of having mental distress, somatic symptoms, anxiety/insomnia or depression

	Unadjusted OR (95% CI)	*P* value	Adjusted OR* (95% CI)	*P* value
Overall mental health score	0.80 (0.70–0.91)	0.001	0.74 (0.64–0.87)	< 0.001
Somatic symptoms	0.93 (0.83–1.04)	0.222	0.88 (0.77–1.01)	0.062
Anxiety/insomnia	0.82 (0.73–0.91)	< 0.001	0.76 (0.66–0.87)	< 0.001
Social dysfunction	N/A^#^			
Depression	0.74 (0.66–0.83)	< 0.001	0.70 (0.60–0.80)	< 0.001

95% CI = 95% confidence interval; Adjusted R^2^ = 38.4%

^#^ 100% of participants have the subscale score at the cut-off point or higher for having social dysfunction

*adjusted for age, age of marriage, education status, occupation status, adequacy of income, number of pregnancies and history of ectopic pregnancy of participants, and spouse’s age and education

## Discussion

The results of our study showed that more than three-fourths of women with EP had mental distress, which was found to be negatively associated with self-esteem of these women. In a clinical trial in Iran [38] examining the Progressive Muscle Relaxation Technique (PMRT), the mean pre-intervention score of GHQ-28 in primigravida women in the first trimester of low-risk pregnancy was comparable to the GHQ-28 score in our study, indicating that mental distress is prevalent among Iranian pregnant women in general. In other studies in Iran, less than 50% of people older than 60 years [39] and 45% of nurses participating in the studies reported to have mental distress [40], compared to 76% of participants in our study reporting mental distress. On the other hand, research in the Northern England Urban community [41] showed that the total mean GHQ-28 score among 273 women in early pregnancy was much lower than that among women with EP in our study.

In our study, self-esteem was measured using the Rosenberg Self-Esteem Scale (RSES), and with a mean RSES score of 4.5 among study participants, their self-esteem was found to be slightly lower compared to other studies among Iranian women. A similar RSES score was observed in a clinical trial with Iranian women with EP reporting the mean baseline RSES score (SD) of 4.4 (4.3) in the control group. However, the RSES score (SD) of 5.1 (3.9) reported in the intervention group was slightly higher than that in our study [42]. Likewise, in a study among breastfeeding mothers in Iran, the mean RSES score (SD) was 5.8 (4.0) [43], which is higher than the RSES score in our study.

Previous research has shown that psychological and somatic symptoms can occur following the loss of pregnancy and that the occurrence of psychological and somatic symptoms is higher in pregnancy loss or EP compared to normal pregnancy. The odds of moderate and severe depression (measured by The Major Depression Index (MDI)) among women with recurrent pregnancy loss in Denmark were found to be 5 times higher than those in women with normal pregnancy, and the incidence of self-reported stress was also higher in women with pregnancy loss [19]. A prospective cohort study among women with early pregnancy loss in London also found that at 1 month and 3 months after experiencing abortion or EP, 28% and 39% of the women respectively, fit into diagnostic criteria for post-traumatic stress disorder. That study also showed that women with EP suffered from higher levels of emotional stress, and were more likely to have symptoms of post-traumatic stress disorder, anxiety and depression than women who have experienced a miscarriage [44]. Findings from our study also suggest that the prevalence of mental distress (as measured by GHQ-28 instrument) in Iranian women with ectopic pregnancy is higher than that in the Iranian women in the general population (37.9%) [45].

In this study, there was a negative correlation between self-esteem and mental health. The relationship of self-esteem with depression and anxiety is well-established in a variety of populations including college students, pregnant women, mothers, and other convenience and clinical samples [46]. However, a birth cohort study in New Zealand showed that low self-esteem scores were associated with a higher risk of mental health problems, although this relationship can be explained better with family and childhood factors (such as socioeconomic background and family functioning) rather than simply the effect of self-esteem [47]. Nevertheless, there is scant research on the relationship between self-esteem and mental health in pregnancy in particular [26, 28], and none in pregnancy loss or ectopic pregnancy. Limited research on this relationship in pregnancy showed that low self-esteem was associated with post-partum depression [28] and that self-esteem is considered one of the most important determinants of psychological problems in pregnancy [26]. Our study has added to the evidence base that this relationship also exists among women with ectopic pregnancy.

Experimental evidence suggests how self-esteem can be improved among women. For instance, a study on the Transactional Analysis training group (eight 90-min weekly sessions) showed that self-esteem of imprisoned women increased from low level to moderate level after attending educational classes [48]. Another study demonstrated the effect of three-component lifestyle intervention [49], that includes cognitive behavioral therapy (CBT) and life-style (food and exercise), on improving self-esteem. Counseling based on health promotion awareness [42] (through reducing ambiguities about EP) was also found to improve self-esteem in women with EP. In addition, a systematic review identified the effectiveness of compassion-focused therapies (CFT) or compassion-based interventions such as a CFT-based neurorehabilitation program, Compassionate Mind Training (CMT) programme, and compassion-focused interventions including general CBT principles and writing about stressful experiences following self-compassionate expressive writing instructions [50]. Previous studies have also shown that self-esteem, self-concept, and self-worth among children and adolescents can be improved through physical activity and exercise such as classroom aerobics with music [51–53]. Given the important role of self-esteem in the mental wellbeing among women who have experienced EP, as evident in our study, having the above services available to them and building their self-esteem during their childhood and adolescence may equip them with the ability to prevent or alleviate mental distress when facing adversities such as EP.

One limitation of our study was the absence of a control group. Previous studies [19, 20, 44] on women with recurrent miscarriage and ectopic pregnancy included women with normal pregnancies as a control group. However, pregnant women might be experiencing specific alternations of mood which makes them a poor control group. In addition, the awareness of being pregnant is likely to affect the individual’s mood, leading to women perhaps being less depressed and anxious than usual, or in some cases the reverse. Another limitation was that causal relationships cannot be established in our study due to its cross-sectional nature. In addition, data were collected after completing the treatment for ectopic pregnancy, immediately before the women were discharged from the hospital, therefore, it is suggested the mental health of these women be assessed after a few weeks, during which natural recovery has run its course.

Our results are relevant to healthcare professionals who provide care to women with EP. If our findings are supported by further studies using longitudinal or experimental designs, consideration should be given to screening of all women who have experienced an ectopic pregnancy for mental distress. Our findings suggest that improving self-esteem may buffer the negative effects of ectopic pregnancy on their mental health. Thus, incorporating assessment of self-esteem and mental health into the care of women with EP might benefit them, and implementation research is needed on how to effectively deliver services for promoting self-esteem and mental wellbeing among those women.

## Conclusion

Mental distress is common among women with ectopic pregnancy. The present study highlights the important role of self-esteem in mental wellbeing among women with ectopic pregnancy, and shows that lower self-esteem is associated with poorer mental health. It is hoped that findings from this study will result in a heightened awareness among health providers to assess and improve self-esteem and mental health in women with ectopic pregnancy.

## Data Availability

The datasets used and analyzed during the current study are available from the corresponding author on reasonable request.

## Abbreviations

SD: Standard deviation
EP: Ectopic pregnancy
IUDs: Intra-uterine devices
OCPs: Oral contraceptives
LNG-EC: Levonorgestrel-emergency contraceptive
RSES: Rosenberg self-esteem scale
GHQ: General health questionnaire
OR: Odds ratio
95% CI: 95% Confidence interval
